# Bis{μ-*cis*-1,3-bis­[(di-*tert*-butyl­phosphan­yl)­oxy]cyclo­hexane-κ^2^ *P*:*P*′}bis­[carbonylnickel(0)] including an unknown solvent molecule

**DOI:** 10.1107/S1600536814007818

**Published:** 2014-04-12

**Authors:** Klara J. Jonasson, Ola F. Wendt

**Affiliations:** a Centre for Analysis and Synthesis, Department of Chemistry, Lund University, PO Box 124, S-221 00 Lund, Sweden

## Abstract

The title compound, [Ni_2_(C_22_H_46_P_2_O_2_)_2_(CO)_2_], is located about a centre of inversion with the Ni^0^ atom within a distorted trigonal–planar geometry. The cyclo­hexyl rings are in the usual chair conformation with the 1,3-*cis* substituents equatorially oriented. No specific inter­molecular inter­actions are noted in the crystal packing. A region of disordered electron density, most probably a disordered deuterobenzene solvent molecule, was treated using the SQUEEZE routine in *PLATON* [Spek (2009 ▶). *Acta Cryst.* D**65**, 148–155]. Its formula mass and unit-cell characteristics were not taken into account during refinement.

## Related literature   

For similar 16-atom macrocyclic dimers with Ni^II^, see: Johnson & Wendt (2011 ▶); Castonguay *et al.* (2008 ▶); Pandarus *et al.* (2008 ▶). For 16-atom macrocyclic dimers of Pd^II^ and Pt^II^ with *cis*-1,3-bis-(di-alkyl­phosphinito)cyclo­hexane ligands, see: Sjövall *et al.* (2001 ▶) and Olsson *et al.* (2007 ▶), respectively. For other examples of Ni^0^ atoms adopting a close to trigonal–planar geometry, see: Rosenthal *et al.* (1990 ▶); Maciejewski *et al.* (2004 ▶); Brun *et al.* (2013 ▶). For an example of a carbon monoxide-induced reductive elimination from a PNP pincer-supported Ni^II^ hydride complex to form a tetra­hedral Ni^0^ dicarbonyl species (PNP = [N(2-P*R*_2_-C_6_H_3_)_2_]^−^), see: Liang *et al.* (2012 ▶).
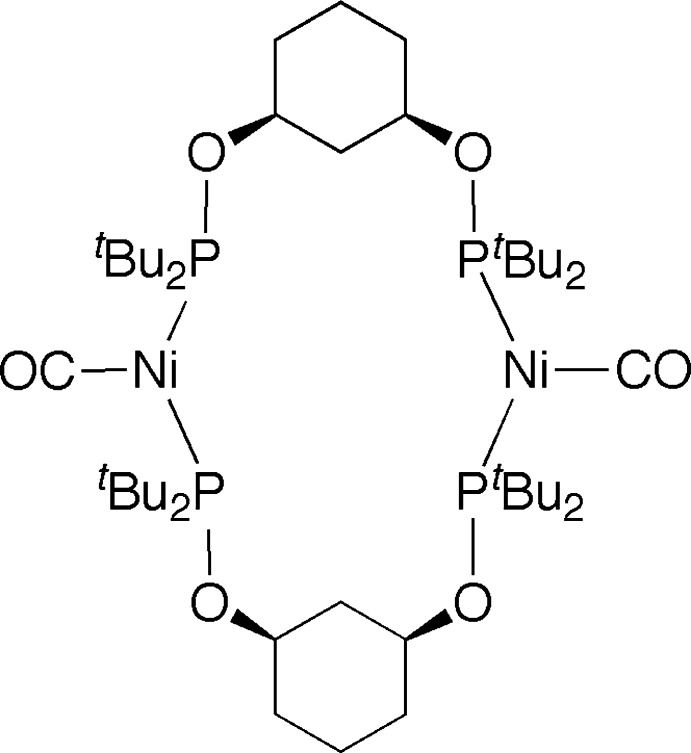


## Experimental   

### 

#### Crystal data  

[Ni_2_(C_22_H_46_O_2_P_2_)_2_(CO)_2_]
*M* *_r_* = 982.50
Monoclinic, 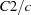

*a* = 31.7851 (9) Å
*b* = 8.5449 (2) Å
*c* = 21.3311 (5) Å
β = 90.995 (2)°
*V* = 5792.7 (3) Å^3^
*Z* = 4
Mo *K*α radiation
μ = 0.80 mm^−1^
*T* = 120 K
0.20 × 0.15 × 0.05 mm

#### Data collection  

Agilent Xcalibur Sapphire3 diffractometer
Absorption correction: multi-scan (*CrysAlis PRO*; Agilent, 2011 ▶) *T*_min_ = 0.883, *T*_max_ = 1.000
27324 measured reflections
6958 independent reflections
4948 reflections with *I* > 2σ(*I*)
*R*_int_ = 0.073

#### Refinement  

*R*[*F* ^2^ > 2σ(*F* ^2^)] = 0.050
*wR*(*F* ^2^) = 0.125
*S* = 1.09
6958 reflections
263 parameters
H-atom parameters constrained
Δρ_max_ = 0.61 e Å^−3^
Δρ_min_ = −0.46 e Å^−3^

### 

Data collection: *CrysAlis PRO* (Agilent, 2011 ▶); cell refinement: *CrysAlis PRO*; data reduction: *CrysAlis PRO*; program(s) used to solve structure: *SHELXS97* (Sheldrick, 2008 ▶); program(s) used to refine structure: *SHELXL97* (Sheldrick, 2008 ▶); molecular graphics: *CrystalMaker* (CrystalMaker, 2001 ▶); software used to prepare material for publication: *SHELXL97*.

## Supplementary Material

Crystal structure: contains datablock(s) I. DOI: 10.1107/S1600536814007818/tk5304sup1.cif

Structure factors: contains datablock(s) I. DOI: 10.1107/S1600536814007818/tk5304Isup2.hkl

CCDC reference: 996025

Additional supporting information:  crystallographic information; 3D view; checkCIF report

## Figures and Tables

**Fig. 1.**
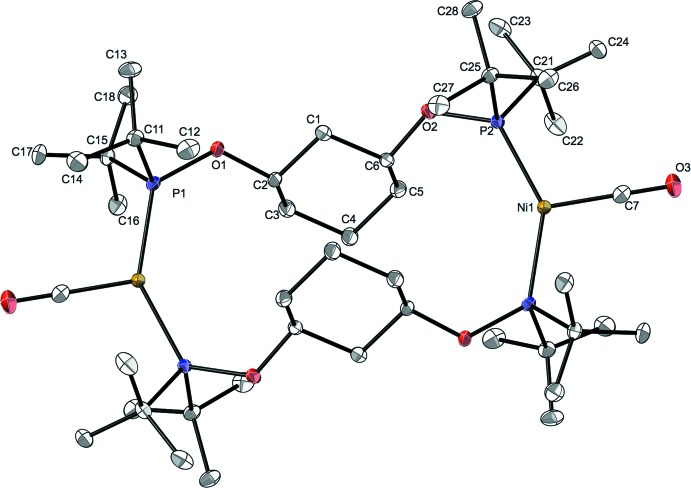
The molecular structure of the centrosymmetric title compound with atom labels and 30% probability displacement ellipsoids. Unlabelled atoms are related by the symmetry operation: 3/2-x, 1/2-y, -z. H-atoms are omitted for clarity.

## supplementary crystallographic information

### 2. Structural commentary

The title compound is formed through a carbon monoxide induced dimerization of a previously synthesized POCOP pincer Ni^II^ hydride complex. The course of the reaction is likely to proceed *via* a reductive elimination of a C—H bond between the metallated carbon and the hydride ligand. In the absence of carbon monoxide the POCOP pincer Ni^II^ hydride complex is stable towards reductive elimination in solution, even at 80 °C and upon addition of 1 eq. di­phenyl­acetyl­ene. Tricoordinate nickel(0) species are coordinately unsaturated, and the steric bulk of the *tert*-butyl substituents on the phospho­rus atoms is likely to have a crucial stabilizing impact on the title compound. It decomposes over a period of hours upon exposure to air.

The title compound has a low solubility in C_6_D_6_ and attempts to obtain ^1^H– and ^13^C-NMR spectra has been unsatisfactory. Dissolving the red crystals of the title compound in CDCl_3_ results in a yellow/green solution and decomposition to several compounds, as indicated by ^31^P-NMR spectroscopy; none was successfully isolated or characterized.

### 5. Synthesis and crystallization

A C_6_D_6_ solution of the compound *trans*-[NiH{*cis*-1,3-Bis-(di-*tert*-butyl­phosphinito) cyclo­hexane}] (10.0 mg, 0.021 mmol) was degassed with repeated freeze-pump-thaw cycles, before addition of CO (3 atm, 0.2 mmol, 10 eq.). Upon standing at room temperature the solution turned gradually darker, and within 48 h deep-red crystals of bis­[µ-[*cis*-1,3-bis­[(di-*tert*-butyl)­phosphinito]cyclo­hexane]-κ^2^-P,*P*']- bis­[carbonyl­nickel(0)] were formed. These were used directly in the X-ray diffraction experiment, but were dried in high-vacuum prior to the elemental analysis. Yield: 8.7 mg (82%). ^31^P{1H} NMR: (202.3 MHz, C_6_D_6_) δ: 177.8 (*s*). Anal. Calcd for C_46_H_92_Ni_2_O_6_P_4_ (982.52): C 56.23, H 9.44. Found: C 56.02, H 9.47.

### 6. Refinement

The H atoms were positioned geometrically and treated as riding on their parent atoms with C—H distances of 0.96–0.98 Å, and with *U*_iso_(H) = 1.2–1.5 *U*_eq_. The asymmetric unit contains half a molecule of the title complex and half a molecule of benzene but this could not be modelled successfully. Solvent contributions were therefore removed from the diffraction data with *PLATON* using the SQUEEZE procedure (Spek, 2009). SQUEEZE estimated the electron count in the void volume of 680 Å^3^ to be 140 which is in reasonable agreement with a total number of four benzene molecules in the unit cell.

### Crystal data

**Table:**

[Ni_2_(C_22_H_46_O_2_P_2_)_2_(CO)_2_]	*F*(000) = 2128
*M**_r_* = 982.50	*D*_x_ = 1.127 Mg m^−^^3^
Monoclinic, *C*2/*c*	Mo *K*α radiation, λ = 0.71073 Å
Hall symbol: -C 2yc	Cell parameters from 6436 reflections
*a* = 31.7851 (9) Å	θ = 2.5–29.1°
*b* = 8.5449 (2) Å	µ = 0.80 mm^−^^1^
*c* = 21.3311 (5) Å	*T* = 120 K
β = 90.995 (2)°	Plates, red
*V* = 5792.7 (3) Å^3^	0.2 × 0.15 × 0.05 mm
*Z* = 4	

### Data collection

**Table:**

Agilent Xcalibur Sapphire3 diffractometer	6958 independent reflections
Radiation source: Enhance (Mo) X-ray Source	4948 reflections with *I* > 2σ(*I*)
Graphite monochromator	*R*_int_ = 0.073
Detector resolution: 16.1829 pixels mm^-1^	θ_max_ = 29.1°, θ_min_ = 2.5°
ω scans	*h* = −42→35
Absorption correction: multi-scan (*CrysAlis PRO*; Agilent, 2011)	*k* = −11→11
*T*_min_ = 0.883, *T*_max_ = 1.000	*l* = −26→26
27324 measured reflections	

### Refinement

**Table:**

Refinement on *F*^2^	Primary atom site location: structure-invariant direct methods
Least-squares matrix: full	Secondary atom site location: difference Fourier map
*R*[*F*^2^ > 2σ(*F*^2^)] = 0.050	Hydrogen site location: inferred from neighbouring sites
*wR*(*F*^2^) = 0.125	H-atom parameters constrained
*S* = 1.09	*w* = 1/[σ^2^(*F*_o_^2^) + (0.050*P*)^2^] where *P* = (*F*_o_^2^ + 2*F*_c_^2^)/3
6958 reflections	(Δ/σ)_max_ = 0.001
263 parameters	Δρ_max_ = 0.61 e Å^−^^3^
0 restraints	Δρ_min_ = −0.46 e Å^−^^3^

### Special details

**Table:**

Geometry. All e.s.d.'s (except the e.s.d. in the dihedral angle between two l.s. planes) are estimated using the full covariance matrix. The cell e.s.d.'s are taken into account individually in the estimation of e.s.d.'s in distances, angles and torsion angles; correlations between e.s.d.'s in cell parameters are only used when they are defined by crystal symmetry. An approximate (isotropic) treatment of cell e.s.d.'s is used for estimating e.s.d.'s involving l.s. planes.
Refinement. Refinement of *F*^2^ against ALL reflections. The weighted *R*-factor *wR* and goodness of fit *S* are based on *F*^2^, conventional *R*-factors *R* are based on *F*, with *F* set to zero for negative *F*^2^. The threshold expression of *F*^2^ > σ(*F*^2^) is used only for calculating *R*-factors(gt) *etc*. and is not relevant to the choice of reflections for refinement. *R*-factors based on *F*^2^ are statistically about twice as large as those based on *F*, and *R*- factors based on ALL data will be even larger.

### Fractional atomic coordinates and isotropic or equivalent isotropic displacement parameters (Å^2^)

**Table:**

	*x*	*y*	*z*	*U*_iso_*/*U*_eq_	
Ni1	0.882994 (11)	0.29002 (4)	0.082823 (15)	0.01812 (11)	
P1	0.83832 (2)	0.33932 (7)	0.15811 (3)	0.01714 (16)	
O1	0.78777 (6)	0.29472 (19)	0.16116 (8)	0.0207 (4)	
C1	0.78065 (8)	0.2143 (3)	−0.11259 (12)	0.0203 (6)	
H1A	0.7814	0.1012	−0.1089	0.024*	
H1B	0.7917	0.2423	−0.1532	0.024*	
P2	0.88701 (2)	0.22270 (7)	−0.01654 (3)	0.01594 (15)	
O2	0.85052 (6)	0.2342 (2)	−0.07197 (8)	0.0218 (4)	
C2	0.73525 (8)	0.2701 (3)	−0.10868 (12)	0.0196 (6)	
H2	0.7231	0.2309	−0.0698	0.023*	
O3	0.96746 (7)	0.3500 (3)	0.13009 (11)	0.0510 (6)	
C3	0.73284 (9)	0.4468 (3)	−0.10912 (13)	0.0264 (6)	
H3A	0.7038	0.4791	−0.1042	0.032*	
H3B	0.7423	0.4857	−0.1492	0.032*	
C4	0.75974 (9)	0.5172 (3)	−0.05680 (14)	0.0280 (7)	
H4A	0.7487	0.4860	−0.0166	0.034*	
H4B	0.7587	0.6304	−0.0594	0.034*	
C5	0.80545 (9)	0.4621 (3)	−0.06177 (13)	0.0249 (6)	
H5A	0.8172	0.5017	−0.1003	0.030*	
H5B	0.8219	0.5041	−0.0269	0.030*	
C6	0.80822 (8)	0.2861 (3)	−0.06116 (12)	0.0194 (6)	
H6	0.7991	0.2472	−0.0204	0.023*	
C7	0.93363 (11)	0.3218 (3)	0.11184 (14)	0.0321 (7)	
C11	0.83370 (9)	0.5570 (3)	0.16926 (12)	0.0226 (6)	
C12	0.80724 (11)	0.6148 (3)	0.11321 (14)	0.0341 (7)	
H12A	0.7798	0.5681	0.1144	0.051*	
H12B	0.8207	0.5858	0.0750	0.051*	
H12C	0.8046	0.7266	0.1152	0.051*	
C13	0.81175 (10)	0.6059 (3)	0.22944 (13)	0.0326 (7)	
H13A	0.7845	0.5579	0.2308	0.049*	
H13B	0.8087	0.7177	0.2302	0.049*	
H13C	0.8283	0.5729	0.2651	0.049*	
C14	0.87701 (10)	0.6353 (3)	0.16623 (15)	0.0342 (7)	
H14A	0.8942	0.6012	0.2011	0.051*	
H14B	0.8737	0.7469	0.1679	0.051*	
H14C	0.8903	0.6069	0.1278	0.051*	
C15	0.85457 (9)	0.2395 (3)	0.23398 (12)	0.0226 (6)	
C16	0.86514 (10)	0.0712 (3)	0.21485 (13)	0.0303 (7)	
H16A	0.8870	0.0725	0.1844	0.045*	
H16B	0.8405	0.0221	0.1971	0.045*	
H16C	0.8745	0.0135	0.2511	0.045*	
C17	0.89435 (10)	0.3151 (3)	0.26297 (13)	0.0287 (7)	
H17A	0.8883	0.4209	0.2748	0.043*	
H17B	0.9164	0.3146	0.2328	0.043*	
H17C	0.9032	0.2567	0.2994	0.043*	
C18	0.81986 (10)	0.2332 (3)	0.28269 (13)	0.0317 (7)	
H18A	0.8128	0.3376	0.2954	0.048*	
H18B	0.8296	0.1747	0.3185	0.048*	
H18C	0.7954	0.1833	0.2648	0.048*	
C21	0.92859 (9)	0.3402 (3)	−0.05827 (13)	0.0227 (6)	
C22	0.92236 (10)	0.5106 (3)	−0.03761 (16)	0.0352 (8)	
H22A	0.9251	0.5175	0.0072	0.053*	
H22B	0.8948	0.5455	−0.0505	0.053*	
H22C	0.9433	0.5755	−0.0566	0.053*	
C23	0.92422 (10)	0.3335 (3)	−0.12995 (14)	0.0329 (7)	
H23A	0.8962	0.3636	−0.1424	0.049*	
H23B	0.9296	0.2288	−0.1440	0.049*	
H23C	0.9441	0.4039	−0.1483	0.049*	
C24	0.97330 (9)	0.2906 (3)	−0.03876 (14)	0.0277 (6)	
H24A	0.9762	0.2945	0.0061	0.042*	
H24B	0.9933	0.3604	−0.0572	0.042*	
H24C	0.9784	0.1858	−0.0530	0.042*	
C25	0.89841 (9)	0.0076 (3)	−0.02237 (12)	0.0212 (6)	
C26	0.93066 (10)	−0.0421 (3)	0.02756 (13)	0.0306 (7)	
H26A	0.9572	0.0072	0.0195	0.046*	
H26B	0.9340	−0.1537	0.0265	0.046*	
H26C	0.9211	−0.0110	0.0682	0.046*	
C27	0.85646 (10)	−0.0734 (3)	−0.00816 (14)	0.0317 (7)	
H27A	0.8357	−0.0441	−0.0393	0.047*	
H27B	0.8472	−0.0418	0.0325	0.047*	
H27C	0.8603	−0.1848	−0.0088	0.047*	
C28	0.91206 (10)	−0.0450 (3)	−0.08764 (13)	0.0287 (7)	
H28A	0.8912	−0.0139	−0.1182	0.043*	
H28B	0.9150	−0.1568	−0.0882	0.043*	
H28C	0.9385	0.0028	−0.0973	0.043*	

### Atomic displacement parameters (Å^2^)

**Table:**

	*U*^11^	*U*^22^	*U*^33^	*U*^12^	*U*^13^	*U*^23^
Ni1	0.0181 (2)	0.01832 (18)	0.01790 (19)	0.00151 (13)	−0.00099 (14)	−0.00328 (12)
P1	0.0194 (4)	0.0159 (3)	0.0160 (3)	0.0014 (3)	−0.0019 (3)	−0.0026 (2)
O1	0.0177 (10)	0.0275 (10)	0.0168 (9)	−0.0013 (8)	−0.0016 (8)	−0.0021 (7)
C1	0.0189 (15)	0.0226 (13)	0.0195 (13)	0.0009 (11)	0.0016 (11)	0.0035 (10)
P2	0.0155 (4)	0.0157 (3)	0.0166 (3)	0.0024 (3)	−0.0002 (3)	0.0007 (2)
O2	0.0164 (10)	0.0310 (10)	0.0179 (9)	0.0043 (8)	−0.0004 (8)	0.0011 (7)
C2	0.0185 (15)	0.0272 (14)	0.0130 (12)	−0.0007 (11)	−0.0015 (11)	0.0003 (10)
O3	0.0249 (14)	0.0843 (18)	0.0434 (15)	−0.0081 (13)	−0.0078 (11)	−0.0117 (12)
C3	0.0228 (16)	0.0263 (14)	0.0299 (16)	0.0042 (12)	−0.0022 (13)	−0.0033 (11)
C4	0.0252 (17)	0.0259 (14)	0.0328 (16)	0.0066 (12)	−0.0059 (13)	−0.0069 (11)
C5	0.0227 (16)	0.0273 (14)	0.0246 (14)	0.0012 (12)	−0.0034 (12)	−0.0039 (11)
C6	0.0168 (15)	0.0271 (14)	0.0141 (12)	0.0027 (11)	−0.0002 (11)	0.0019 (10)
C7	0.0313 (19)	0.0401 (17)	0.0249 (16)	−0.0002 (14)	−0.0007 (14)	−0.0080 (12)
C11	0.0256 (16)	0.0165 (12)	0.0257 (14)	0.0024 (11)	0.0002 (12)	−0.0032 (10)
C12	0.047 (2)	0.0236 (15)	0.0315 (17)	0.0094 (14)	−0.0019 (15)	0.0035 (12)
C13	0.042 (2)	0.0256 (15)	0.0302 (16)	0.0111 (14)	0.0045 (14)	−0.0059 (12)
C14	0.0341 (19)	0.0184 (13)	0.050 (2)	−0.0020 (13)	0.0053 (15)	−0.0072 (12)
C15	0.0227 (16)	0.0261 (14)	0.0188 (13)	0.0022 (12)	−0.0047 (11)	0.0017 (10)
C16	0.0355 (19)	0.0226 (14)	0.0325 (16)	0.0049 (13)	−0.0088 (14)	0.0020 (11)
C17	0.0278 (18)	0.0333 (16)	0.0247 (15)	0.0007 (13)	−0.0099 (13)	−0.0024 (11)
C18	0.0328 (19)	0.0401 (17)	0.0221 (15)	0.0033 (14)	−0.0012 (13)	0.0046 (12)
C21	0.0180 (15)	0.0223 (13)	0.0278 (15)	0.0008 (11)	0.0010 (12)	0.0032 (11)
C22	0.0286 (18)	0.0213 (14)	0.056 (2)	−0.0025 (13)	0.0063 (15)	0.0081 (13)
C23	0.0265 (18)	0.0400 (17)	0.0326 (17)	0.0019 (14)	0.0078 (14)	0.0152 (13)
C24	0.0205 (16)	0.0283 (15)	0.0343 (16)	0.0002 (12)	0.0023 (13)	0.0002 (11)
C25	0.0267 (16)	0.0163 (12)	0.0204 (13)	0.0015 (11)	−0.0003 (12)	−0.0013 (10)
C26	0.042 (2)	0.0225 (14)	0.0273 (15)	0.0094 (13)	−0.0057 (14)	0.0014 (11)
C27	0.039 (2)	0.0168 (13)	0.0390 (18)	−0.0041 (13)	0.0057 (15)	−0.0002 (12)
C28	0.042 (2)	0.0203 (13)	0.0242 (15)	0.0059 (13)	−0.0012 (13)	−0.0048 (11)

### Geometric parameters (Å, º)

**Table:**

Ni1—C7	1.736 (3)	C14—H14B	0.9600
Ni1—P2	2.2021 (7)	C14—H14C	0.9600
Ni1—P1	2.2028 (7)	C15—C18	1.530 (4)
P1—O1	1.654 (2)	C15—C16	1.534 (4)
P1—C11	1.882 (2)	C15—C17	1.540 (4)
P1—C15	1.893 (3)	C16—H16A	0.9600
O1—C2^i^	1.438 (3)	C16—H16B	0.9600
C1—C6	1.521 (4)	C16—H16C	0.9600
C1—C2	1.523 (4)	C17—H17A	0.9600
C1—H1A	0.9700	C17—H17B	0.9600
C1—H1B	0.9700	C17—H17C	0.9600
P2—O2	1.6448 (19)	C18—H18A	0.9600
P2—C25	1.878 (2)	C18—H18B	0.9600
P2—C21	1.894 (3)	C18—H18C	0.9600
O2—C6	1.438 (3)	C21—C24	1.534 (4)
C2—O1^i^	1.438 (3)	C21—C23	1.534 (4)
C2—C3	1.512 (3)	C21—C22	1.536 (4)
C2—H2	0.9800	C22—H22A	0.9600
O3—C7	1.162 (4)	C22—H22B	0.9600
C3—C4	1.518 (4)	C22—H22C	0.9600
C3—H3A	0.9700	C23—H23A	0.9600
C3—H3B	0.9700	C23—H23B	0.9600
C4—C5	1.533 (4)	C23—H23C	0.9600
C4—H4A	0.9700	C24—H24A	0.9600
C4—H4B	0.9700	C24—H24B	0.9600
C5—C6	1.507 (3)	C24—H24C	0.9600
C5—H5A	0.9700	C25—C26	1.526 (4)
C5—H5B	0.9700	C25—C28	1.533 (4)
C6—H6	0.9800	C25—C27	1.537 (4)
C11—C13	1.530 (4)	C26—H26A	0.9600
C11—C12	1.532 (4)	C26—H26B	0.9600
C11—C14	1.533 (4)	C26—H26C	0.9600
C12—H12A	0.9600	C27—H27A	0.9600
C12—H12B	0.9600	C27—H27B	0.9600
C12—H12C	0.9600	C27—H27C	0.9600
C13—H13A	0.9600	C28—H28A	0.9600
C13—H13B	0.9600	C28—H28B	0.9600
C13—H13C	0.9600	C28—H28C	0.9600
C14—H14A	0.9600		
			
C7—Ni1—P2	108.38 (10)	C11—C14—H14C	109.5
C7—Ni1—P1	108.39 (10)	H14A—C14—H14C	109.5
P2—Ni1—P1	143.19 (3)	H14B—C14—H14C	109.5
O1—P1—C11	98.33 (11)	C18—C15—C16	108.2 (2)
O1—P1—C15	96.52 (11)	C18—C15—C17	109.8 (2)
C11—P1—C15	110.98 (12)	C16—C15—C17	108.5 (2)
O1—P1—Ni1	128.58 (7)	C18—C15—P1	114.0 (2)
C11—P1—Ni1	109.51 (9)	C16—C15—P1	104.64 (18)
C15—P1—Ni1	111.55 (9)	C17—C15—P1	111.38 (18)
C2^i^—O1—P1	122.67 (16)	C15—C16—H16A	109.5
C6—C1—C2	111.6 (2)	C15—C16—H16B	109.5
C6—C1—H1A	109.3	H16A—C16—H16B	109.5
C2—C1—H1A	109.3	C15—C16—H16C	109.5
C6—C1—H1B	109.3	H16A—C16—H16C	109.5
C2—C1—H1B	109.3	H16B—C16—H16C	109.5
H1A—C1—H1B	108.0	C15—C17—H17A	109.5
O2—P2—C25	98.35 (11)	C15—C17—H17B	109.5
O2—P2—C21	96.88 (11)	H17A—C17—H17B	109.5
C25—P2—C21	110.52 (12)	C15—C17—H17C	109.5
O2—P2—Ni1	128.64 (7)	H17A—C17—H17C	109.5
C25—P2—Ni1	109.50 (8)	H17B—C17—H17C	109.5
C21—P2—Ni1	111.50 (9)	C15—C18—H18A	109.5
C6—O2—P2	123.55 (15)	C15—C18—H18B	109.5
O1^i^—C2—C3	110.8 (2)	H18A—C18—H18B	109.5
O1^i^—C2—C1	107.85 (19)	C15—C18—H18C	109.5
C3—C2—C1	111.1 (2)	H18A—C18—H18C	109.5
O1^i^—C2—H2	109.0	H18B—C18—H18C	109.5
C3—C2—H2	109.0	C24—C21—C23	109.1 (2)
C1—C2—H2	109.0	C24—C21—C22	107.9 (2)
C2—C3—C4	111.3 (2)	C23—C21—C22	108.1 (2)
C2—C3—H3A	109.4	C24—C21—P2	112.14 (18)
C4—C3—H3A	109.4	C23—C21—P2	113.38 (19)
C2—C3—H3B	109.4	C22—C21—P2	105.86 (19)
C4—C3—H3B	109.4	C21—C22—H22A	109.5
H3A—C3—H3B	108.0	C21—C22—H22B	109.5
C3—C4—C5	110.5 (2)	H22A—C22—H22B	109.5
C3—C4—H4A	109.6	C21—C22—H22C	109.5
C5—C4—H4A	109.6	H22A—C22—H22C	109.5
C3—C4—H4B	109.6	H22B—C22—H22C	109.5
C5—C4—H4B	109.6	C21—C23—H23A	109.5
H4A—C4—H4B	108.1	C21—C23—H23B	109.5
C6—C5—C4	111.2 (2)	H23A—C23—H23B	109.5
C6—C5—H5A	109.4	C21—C23—H23C	109.5
C4—C5—H5A	109.4	H23A—C23—H23C	109.5
C6—C5—H5B	109.4	H23B—C23—H23C	109.5
C4—C5—H5B	109.4	C21—C24—H24A	109.5
H5A—C5—H5B	108.0	C21—C24—H24B	109.5
O2—C6—C5	111.2 (2)	H24A—C24—H24B	109.5
O2—C6—C1	106.77 (19)	C21—C24—H24C	109.5
C5—C6—C1	111.3 (2)	H24A—C24—H24C	109.5
O2—C6—H6	109.2	H24B—C24—H24C	109.5
C5—C6—H6	109.2	C26—C25—C28	110.8 (2)
C1—C6—H6	109.2	C26—C25—C27	108.1 (2)
O3—C7—Ni1	176.8 (3)	C28—C25—C27	107.9 (2)
C13—C11—C12	108.3 (2)	C26—C25—P2	110.76 (18)
C13—C11—C14	109.8 (2)	C28—C25—P2	113.90 (17)
C12—C11—C14	107.9 (2)	C27—C25—P2	104.98 (18)
C13—C11—P1	114.45 (18)	C25—C26—H26A	109.5
C12—C11—P1	105.24 (18)	C25—C26—H26B	109.5
C14—C11—P1	110.74 (18)	H26A—C26—H26B	109.5
C11—C12—H12A	109.5	C25—C26—H26C	109.5
C11—C12—H12B	109.5	H26A—C26—H26C	109.5
H12A—C12—H12B	109.5	H26B—C26—H26C	109.5
C11—C12—H12C	109.5	C25—C27—H27A	109.5
H12A—C12—H12C	109.5	C25—C27—H27B	109.5
H12B—C12—H12C	109.5	H27A—C27—H27B	109.5
C11—C13—H13A	109.5	C25—C27—H27C	109.5
C11—C13—H13B	109.5	H27A—C27—H27C	109.5
H13A—C13—H13B	109.5	H27B—C27—H27C	109.5
C11—C13—H13C	109.5	C25—C28—H28A	109.5
H13A—C13—H13C	109.5	C25—C28—H28B	109.5
H13B—C13—H13C	109.5	H28A—C28—H28B	109.5
C11—C14—H14A	109.5	C25—C28—H28C	109.5
C11—C14—H14B	109.5	H28A—C28—H28C	109.5
H14A—C14—H14B	109.5	H28B—C28—H28C	109.5

Symmetry code: (i) −*x*+3/2, −*y*+1/2, −*z*.
